# Central Sleep Apnea Syndrome Can Complicate Neuromyelitis Optica Spectrum Disorder: A Case Report

**DOI:** 10.3389/fped.2020.547474

**Published:** 2020-09-25

**Authors:** Céline Morelli, Alec Aeby, Sonia Scaillet, Grammatina Boitsios, Daphné Vens, Cynthia Prigogine, Dominique Biarent, Alfredo Vicinanza

**Affiliations:** ^1^Department of Pediatrics, Hôpital Universitaire des Enfants Reine Fabiola, Université Libre de Bruxelles (ULB), Brussels, Belgium; ^2^Division of Pediatric Neurology, Hôpital Universitaire des Enfants Reine Fabiola, Université Libre de Bruxelles (ULB), Brussels, Belgium; ^3^Department of Sleep Medicine, Hôpital Universitaire des Enfants Reine Fabiola, Université Libre de Bruxelles (ULB), Brussels, Belgium; ^4^Department of Pediatric Medical Imaging, Hôpital Universitaire des Enfants Reine Fabiola, Université Libre de Bruxelles (ULB), Brussels, Belgium; ^5^Pediatric Intensive Care Unit, Hôpital Universitaire des Enfants Reine Fabiola, Université Libre de Bruxelles (ULB), Brussels, Belgium; ^6^Department of Chronic Noninvasive Ventilation, Hôpital Universitaire des Enfants Reine Fabiola, Université Libre de Bruxelles (ULB), Brussels, Belgium

**Keywords:** neuromyelitis optica spectrum disorder, central sleep apnea syndrome, area postrema syndrome, children, noninvasive ventilation, sleep-disordered breathing, case report

## Abstract

Neuromyelitis optica spectrum disorder is a rare, relapsing autoimmune disease of the central nervous system. Various initial presentations can delay diagnosis and treatment. A 14-year-old girl was admitted to the emergency department owing to respiratory insufficiency. Repeated history-taking and neuroimaging revealed an area postrema syndrome. A diagnosis of neuromyelitis optica spectrum disorder with positive aquaporin-4 antibodies has finally been established. The patient improved significantly with immunosuppressive therapy. However, her 3-year follow-up still showed sleep-disordered breathing requiring nocturnal bilevel positive airway pressure therapy. We report an original case of NMOSD leading to persistent central sleep apnea syndrome.

## Introduction

Neuromyelitis optica spectrum disorder (NMOSD) is a rare autoimmune astrocytopathy (1). NMOSD presents with optic neuritis and transverse myelitis but also diencephalic, brainstem and cerebral hemisphere lesions (2). Patients may have monophasic or recurrent evolution and develop a wide spectrum of neurologic manifestations that can lead to substantial disabilities and even death (1). In 2004, highly specific serum autoantibodies against the aquaporin-4 water channel (AQP4-IgG) expressed on astrocytes were discovered (1). In 2015, the International Panel for NMO Diagnosis developed revised diagnostic criteria for AQP4-IgG positive and negative patients (3). The reported prevalence of NMOSD ranges between 0.5 and 4 cases per 100,000 people worldwide (4). The average age at diagnosis is 39 years although some pediatric cases have already been described (5).

We report the case of NMOSD in a teenager presenting with respiratory insufficiency at diagnosis and whose evolution led to long-term central sleep apnea syndrome.

## Case Description

A 14-year-old girl was admitted to the emergency department owing to fever, cough and respiratory distress with hypoxemia. Medical and family histories were non-contributory. First investigations showed raised levels of inflammatory markers and pulmonary consolidation. She received low-flow oxygen and intravenous antibiotics because of suspected bacterial pneumonia.

**Figure 1:**
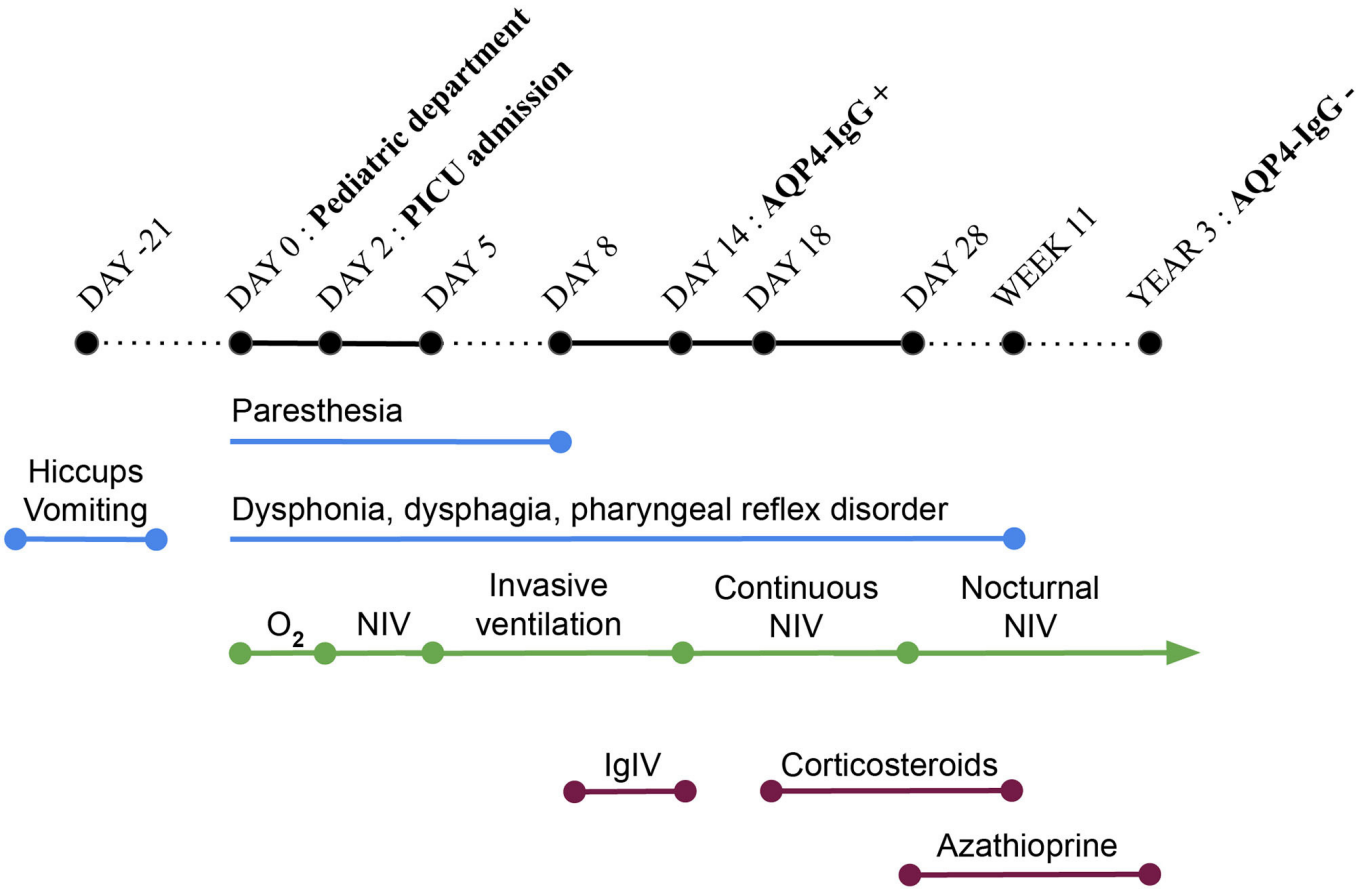
Timeline. PICU, pediatric intensive care unit; O_2_, oxygen; NIV, non-invasive ventilation; IgIV, intravenous immunoglobulin therapy; AQP-IgG, autoantibodies against the aquaporin-4 water channel.

At day 2 after admission, the patient's respiratory status deteriorated (Figure 1). Pulmonary imaging showed bilateral infiltrates, more prominent in the right lung. She was referred to our pediatric intensive care unit for noninvasive ventilation (NIV). A new history-taking revealed that, 3 weeks before admission, the patient presented intractable vomiting and hiccups for 7 days. She also recently complained about abnormal gait, paresthesia and swallowing disorder. A new physical examination showed dysphonia and alteration of the pharyngeal reflex. Cerebrospinal fluid analysis showed moderate lymphocytic pleocytosis treated as infectious meningoencephalitis. However, no pathogens were isolated by extended infectious investigations including bronchoalveolar lavage, blood and cerebrospinal fluid cultures, serological and microbiological testing.

**Figure 2:**
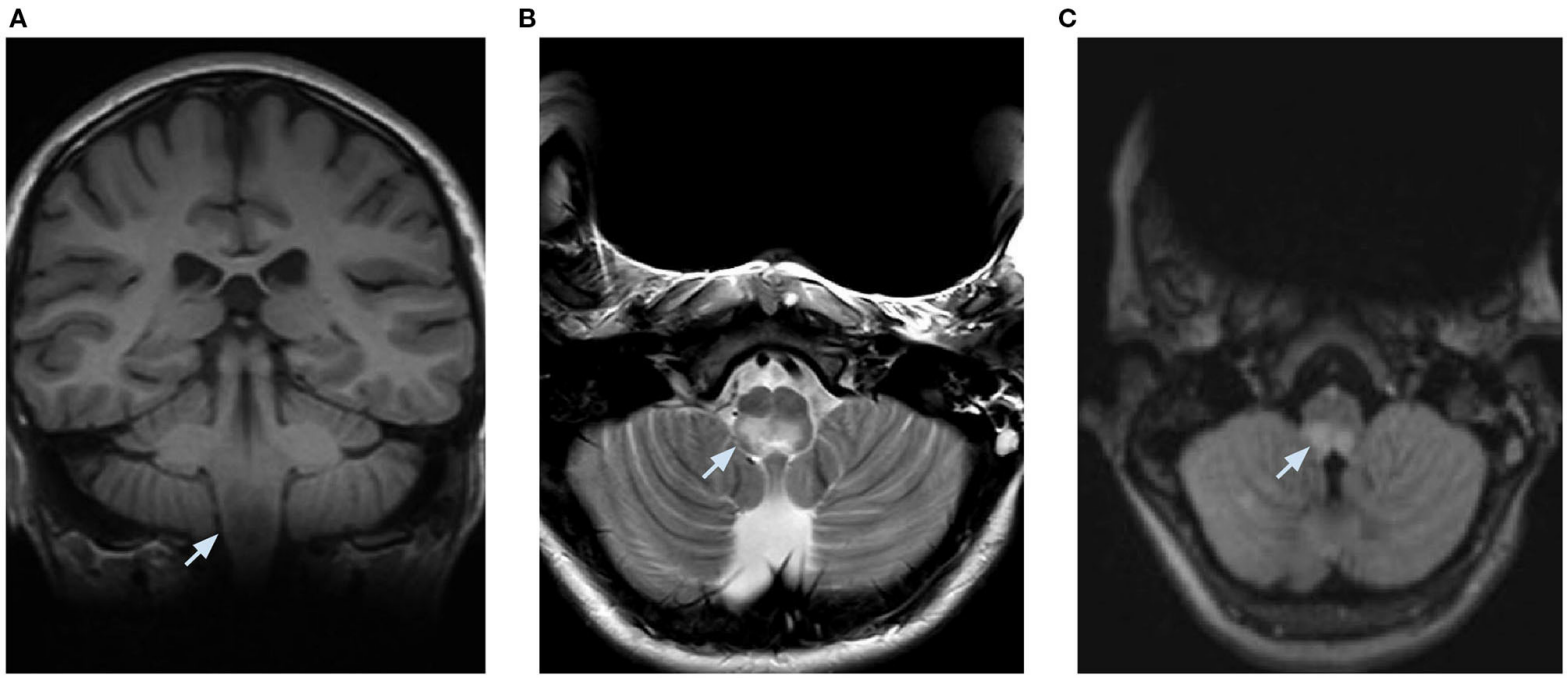
Brain MRI performed at 1.5 Tesla and limited by metallic artifacts. **(A)** coronal T1 weighted imaging showing a low signal intensity, **(B)** axial T2 weighted imaging, and **(C)** axial fluid attenuation inversion recovery showing a high signal intensity in the posterior part of the medulla oblongata bilaterally.

At day 5, a decompensated respiratory insufficiency required mechanical ventilation. A 1.5 Tesla brain magnetic resonance imaging (MRI) showed a high signal intensity in T2 weighted sequence and fluid attenuation inversion recovery and a low signal intensity in T1 weighted imaging without enhancement after the administration of a contrast medium in the dorsal part of the medulla oblongata, where the area postrema is located. Due to metallic artifacts, optic nerves could not be investigated (Figure 2). The MRI of the spinal cord was normal. These findings, along with the clinical history of neurologic deterioration following intractable vomiting and hiccups, were suggestive of the well-known area postrema syndrome.

At day 8, there were no more paresthesia, but the patient still complained with dysphagia, dysphonia and had alteration of the pharyngeal reflex. Because of the high suspicion of an autoimmune disease and the severity of the presentation, high-dose intravenous immunoglobulin therapy was initiated. At day 14, mechanical ventilation was weaned off. Noninvasive ventilation was thereafter introduced. NMOSD was confirmed by detection of AQP4-IgG in blood and cerebrospinal fluid using the indirect immunofluorescence pattern with transfected and control-transfected cells as reacting substrates. The patient still presented some dysphagia and dysphonia. The prolonged need for NIV was thought to be related to brainstem lesions. Because of this severe impairment, a corticosteroid treatment was initiated at day 18, despite the suspected lung infection which was nonetheless under control. A 3-day intravenous high-dose corticosteroid treatment (methylprednisolone 1 g/day) was followed by a long-course oral corticosteroid therapy (methylprednisolone 1 mg/kg/day). Immunosuppression with azathioprine (2 mg/kg/day) was started on day 28 as well. Antibiotic prophylaxis was prescribed because of the iatrogenic immunosuppression.

**Figure 3:**
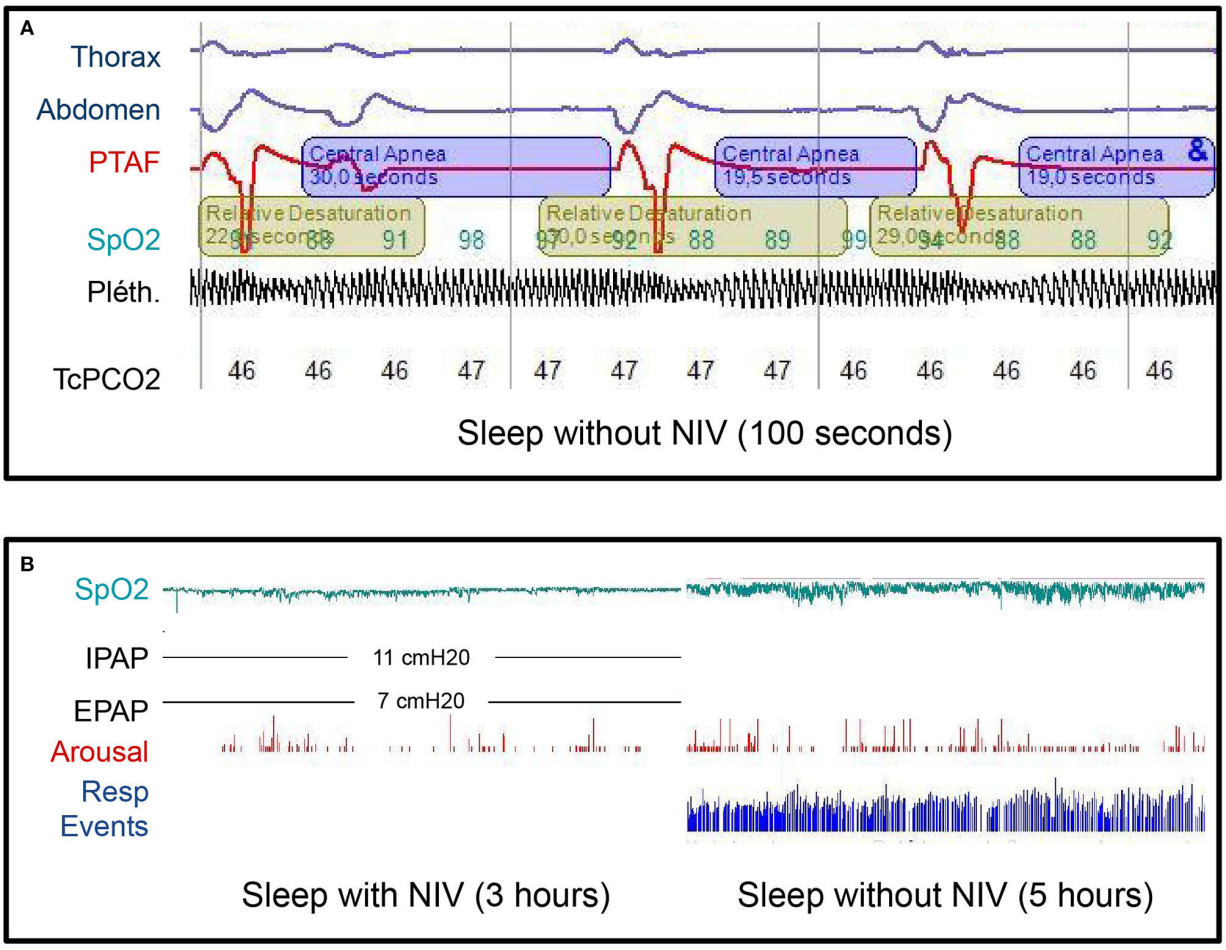
**(A)** Diagnostic sleep study without noninvasive ventilation (NIV) at day 34 after admission. Note three central apnea (cessation of airflow associated with the absence of respiratory efforts and therefore no thoracic or abdominal movements) with subsequent hypoxemia. The apnea-hypopnea index in this study was 110/h. The mean respiratory rate was 3/min. THO, thoracic belt signal; ABD, abdominal belt signal; PTAF, pressure transducer airflow; SpO2, saturation measured by pulse oximetry; Pléth., plethysmography waveform derived from the pulse oximeter; TcPCO_2_, transcutaneous carbon dioxide monitoring. **(B)** Diagnostic sleep study with and without NIV, 3 years after the diagnosis. No respiratory events occurred during the first part of the night with NIV and the SpO2 was above 90% all the time. During the second part of the night without NIV, the apnea-hypopnea index was 110/h and the SpO2 dropped below 90% for 29 min over 5 h with minimal SpO2 of 80%. No recovery of the central sleep apnea syndrome occurred between those two sleep studies. IPAP, inspiratory airway pressure; EPAP, expiratory airway pressure; cmH_2_O, centimeters of water; Resp events, Respiratory events.

At day 28, the patient did not need any respiratory support during the daytime anymore but NIV was still necessary while sleeping. Thus, a polysomnography performed at day 34 revealed central sleep apnea with respiratory rate at 3/min and intermittent hypoxemia. No apnea was observed under NIV in a bilevel positive airway pressure mode (Figure 3A).

The patient was discharged at day 36 with prednisone (1 mg/kg/day), azathioprine (2 mg/kg/day) and nocturnal NIV as home treatment. Dysphagia and dysphonia were completely resolved 2 months after the initiation of the immunosuppressive treatment. The corticosteroid therapy was gradually decreased and stopped 4 months after diagnosis. A brain MRI performed 2 months after discharge showed regression of the lesions. No visual function impairment occurred and optical coherence tomography did not reveal any retinal thinning (6).

The last nocturnal polysomnography at 3-year follow-up was performed with and without NIV. While sleeping without NIV, the patient had a central apnea-hypopnea index = 110/h, a spontaneous respiratory rate = 2–3/min with a mean oxygen saturation measured by pulse oximetry (SpO2) and a nadir SpO2, respectively, at 94 and 80% (Figure 3B). Based upon these results, the nocturnal VNI was maintained. However, at that time, the brain MRI showed a complete recovery of the previous detectable lesions and serum AQP4-IgG were negative. As no clinical relapses had occurred in our 3-year follow-up, azathioprine was discontinued a few weeks before the submission of this case report.

## Discussion

Herein, we describe the acute phase and the three-year follow-up of the first reported case of NMOSD complicated by persistent central sleep apnea syndrome requiring chronic nocturnal NIV. Moreover, this case illustrates that some initial symptoms attributed to the area postrema syndrome (intractable hiccups and vomiting) can be transient and resolve spontaneously whereas some others (absence of the pharyngeal reflex and dysphagia) may persist longer and be underrecognized. Area postrema syndrome is only observed in 12% of AQP4-IgG seropositive patients (7). That was the first clinical manifestation of NMOSD in our patient. However, those symptoms were not spontaneously reported by the patient and her family, thus highlighting the importance of targeted interrogation and complete neurologic examination to help make this kind of diagnosis.

Respiratory insufficiency was the main symptom at the admission of our patient. Although an infectious pneumonia could not be documented, we speculated that repetitive aspirations due to strong hiccups, dysphagia and the absence of pharyngeal reflex have precipitated an aspiration pneumonia. Moreover, the brain lesion exhibited by the patient involved the medulla oblongata, where the dorsal respiratory group of neurons is situated. Therefore, we believe that the respiratory insufficiency requiring long-term ventilation at pediatric intensive care unit might have appeared because of the brainstem dysfunction itself. This hypothesis is better supported by the patient's follow-up, showing persistent central sleep apnea syndrome as long-term neurologic sequela of the NMOSD initial attack.

Currently, very little is known about NMOSD-induced sleep apnea.

To our knowledge, we describe the first case of NMOSD with this kind of severe sleep-disordered breathing. However, disrupted sleep architecture and abnormal polysomnography have already been described in other NMOSD patients (8, 9). Song Y. et al. performed polysomnography in NMOSD and healthy adults in a prospective cross-sectional study. They demonstrated that sleep-disordered breathing was significantly more frequent in the NMOSD group compared to healthy controls (18 vs. 5%, *p* 0.0067). However, polysomnographic studies in the NMOSD group showed a much lower apnea-hypopnea index (maximum 10/h) than that we found in our patient (110/h). Moreover, unlike our patient, this adult cohort presented obstructive sleep apnea and none required any NIV (8).

Because of the high suspicion of autoimmune disease and the pending result of the AQP4-IgG, an intravenous immunoglobulin treatment was first administered to our patient. Immunoglobulin therapy is not the first-line treatment recommended in NMOSD. Although its use has been discussed in NMOSD, there are no randomized controlled trials to establish its efficacy (5).

The patient's neurologic status finally improved a few days after the initiation of steroid infusions (Figure 1) even if that could be due to the natural course of the disease as well.

However, AQP4-IgG-seropositive patients are assumed to be at risk for relapse indefinitely and should be preventively treated (3). Well-established data revealed that, unlike multiple sclerosis, disability in NMOSD occurs as cumulative sequelae of distinct attacks and secondary progressive phase is uncommon (10). Thus, minimizing the frequency and the severity of attacks should be the primary therapeutic goal in NMOSD management (10). Thereby, long-term immunosuppressive therapy with azathioprine was prescribed to our patient and continued for 3 years without any adverse reaction or clinical relapse. Interestingly, in a recent systematic review published after this case occurred, the authors suggested the use of azathioprine as second-line therapy because other immunosuppressants as rituximab and mycophenolate mofetil showed better tolerability and effectiveness (11).

## Conclusion

We described a case of NMOSD in a 14-year-old girl meeting the International Panel for NMO Diagnosis diagnostic criteria. NMOSD can manifest with severe respiratory insufficiency but other less obvious symptoms could delay diagnosis. A prompt recognition of symptoms may lead to better and earlier treatment. Long-term immunosuppressive therapy should be considered to avoid NMOSD relapses.

We found that brainstem lesions in NMOSD might cause persistent central sleep apnea syndrome requiring nocturnal NIV. Clinicians should be aware of this potential complication and sleep disorders should be screened and followed-up after NMOSD attacks.

## Data Availability Statement

The original contributions presented in the study are included in the article/supplementary material, further inquiries can be directed to the corresponding author/s.

## Ethics Statement

Written informed consent was obtained from the parents of the patient for the publication of this case report and any potentially identifying information/images.

## Author Contributions

CM concepted and designed the work, wrote, and structured the manuscript. AV drafted, structured the manuscript, and revised it critically. AA, SS, GB, DV, CP, and DB revised the manuscript. All authors interpreted the data, managed the patient, contributed to the article and approved the submitted version.

## Conflict of Interest

The authors declare that the research was conducted in the absence of any commercial or financial relationships that could be construed as a potential conflict of interest.
